# Knowledge, attitude and practice of health care workers on measuring adult vitamin D level, diagnosis of deficiency, and management of consequent health conditions in three ecologies of Ethiopia: a cross-sectional study

**DOI:** 10.1186/s40795-020-00404-0

**Published:** 2020-12-21

**Authors:** Wubegzier Mekonnen, Yeweyenhareg Feleke, Yakob Desalegn, Getahun Tarekegne, Biruk Lambisso, Jemal Haidar, Tewabech Zewede

**Affiliations:** 1 School of Public Health, College of Health Sciences, Addis Ababa University, Addis Ababa, Ethiopia; 2 School of Medicine, College of Health Sciences, Addis Ababa University, Addis Ababa, Ethiopia; 3 Sante Medical College, Addis Ababa, Ethiopia

**Keywords:** Adult vitamin D deficiency, Ecology, Ethiopia

## Abstract

**Background:**

Vitamin D is essential for health and its shortage exacerbates overall mortality. Health care workers (HCWs) need to educate on its uses and sources although studies indicate their low level of practice. The main aim of this study is therefore to assess the knowledge, attitude and practice of measuring adult vitamin D status, diagnosis of deficiency and managing health consequences among HCWs in Ethiopia.

**Methods:**

This study was conducted in three ecologies covering lowland, midland and highland districts. A total of 405 health care workers with different levels were interviewed. Tablets were used for data collection to archiving in a cloud server. Data were exported to Stata version 14 software for cleaning and analysis. Rates were computed and the Chi-square test was used to compare differences between the two groups. Binary logistic regression was used to measure the strength, direction and significance of the association between different covariates and the practice of HCWs.

**Result:**

The level of knowledge, positive attitude and good practice in measuring adult vitamin D status, diagnosis of deficiency and managing health consequences among HCWs was 210 (51.8%), 261(63.5%) and 195(47.4%) respectively. The odds of good practice in the provision of adult vitamin D service were AOR = 6.87: 95% CI (3.57, 13.21) and AOR = 2.20: 95% CI (1.23, 3.92) times higher among HCWs in Addis Ababa and highlands compared with those working in lowlands. Good practice among clinicians was AOR = 4.26: 95% CI (1.48, 12.25) times higher compared with those working in leadership positions. The likelihood was AOR = 1.96: 95% CI (1.19, 3.23) times higher among those with good knowledge compared with those with poor knowledge. Besides, good practice in adult vitamin D service provision was AOR = 2.30: 95% CI (1.40, 3.78) times higher among those with positive attitude compared with those who had negative attitude.

**Conclusions:**

A little over half of HCWs have good knowledge and close to two-thirds of them have positive attitude while less than half of them have good practice on adult vitamin D deficiency. Besides, HCWs’ residential ecology, clinical position, knowledge and attitude is associated with good practice on adult vitamin D. It is essential to provide rigorous and continuous training for HCWs focusing on their deployment ecology.

## Background

Several studies showed that vitamin D is essential for bone health, extraskeletal tissues, cancer prevention, boosting immune function, infection control, and regulating cell growth, blood pressure, and cardiovascular diseases and its insufficiency has been strongly associated with increased overall mortality [1–3].

Although skin synthesis through direct exposure to ultraviolet B radiation (Direct UVB) is the vital source of vitamin D, it may not sometimes guarantee sufficiency even among residents living in the equator as evidenced from some studies [4–7]. Therefore, there is need for having food items such as fatty fish and fish liver oil that are among the few natural food sources recommended for vitamin D.

The prevalence of adult vitamin D deficiency is increasing even in recent years [6, 7]. The health consequences of a deficiency in vitamin D among adults have also been witnessed in several studies [5, 8, 9]. Health care workers (HCWs) should therefore consistently educate their communities on the uses of vitamin D, its sources, and possible health consequences. However, even recent studies done outside of Ethiopia indicated that the level of knowledge on adult vitamin D deficiency among health care workers has not been as high as expected [10–12].

On one hand, Ethiopia embarked an expansion of health care workers’ training in recent decades to improve its health care delivery system although some of the training programs used expedited and abridged approaches which might compromise the professional competency of health workers [13]. On the other hand, previous studies have also showed that health care workers expressed greater concern about skin cancer which could be caused by excessive exposure to ultraviolet rays than vitamin D deficiency. A study in Australia revealed that there was some confusion in general practice regarding vitamin D, sun exposure, sun protection and skin cancer risk. Some of the pieces of advice that general practitioners are offering may needlessly increase their patients’ risk for vitamin D insufficiency or skin cancer [14–17]. Another study done in New Zealand among general practitioners indicated that concern about the potentially negative impact of skin cancer prevention on vitamin D status may undermine appropriate sun-protective recommendations. Other educational materials also impart knowledge on vitamin D and improve perception about it indicating that significantly less summer sun exposure was required for those with high sun sensitivity to achieve adequate vitamin D, suggesting a potential positive impact of such resources. Accordingly, health education could be targeted towards such health care workers who are less likely to promote existing recommendations [18, 19].

Additional surveys conducted in sub-Saharan Africa and other parts of the world revealed that the level of knowledge, attitude, and practice on adult vitamin D deficiency among health care workers is not as high as expected [20, 21]. However, there is limited information with regards to this issue and no study is conducted which document the factors associated with the practice of vitamin D level measurement, diagnosis of deficiency, and the associated health consequences in the African context which warrants the conduct of such a study in one of the most populous countries with different agro-ecological zones.

It is therefore imperative to explore the competencies of these health care workers in giving health-related service to such specific and very important nutrients as vitamin D. The main aim of this study is, therefore, to measure the level of knowledge, attitude, and practice on the calibration of vitamin D status, diagnosis of deficiency and management and treatment of its health consequences among health care workers deployed in three different ecologies of Ethiopia.

## Methods

Ethiopia is a country with a population of over 105 million persons that has different agro-ecologies including highland, midland, and lowland areas [22]. The practice of sun exposure is strongly associated with the dressing style of people that in turn is related to the religion they confess, culture and economic activities of the society (for instance if they are engaged in outdoor activities or stay indoor most of their time). Dressing invariably differs by residence type (whether people live in rural or urban areas; and they reside in highland, midland, or lowland areas).

A facility-based cross-sectional design was conducted in lowland, midland, and highland areas that were purposefully selected. Addis Ababa was considered as one of the study areas for this study as it reveals the urban life style and represents the midland ecology in the Ethiopian context. *Menze-Gera* district represents the high land area and *Qewot* is a rural lowland district chosen for this study all of which are located in North Shoa zone of Amhara region.

This study enrolled health care workers deployed in the three ecologies and included health extension workers (primary health workers whose main task is disease prevention and promotion with limited curative care services), nurses, health officers, general practitioners and physicians with specialization. The sample size of 424 was calculated using a formula to calculate a single population proportion with the assumption of 50% prevalence, 95% confidence level, 5% margin of error and 10% non-response rate.

The sample size was equally allocated to the three study ecologies. Ten health posts were randomly selected from each study ecology and interviewed all the available health extension workers who were altogether 20 in total. All public hospitals in selected rural lowland and highland study districts were also considered. The number of study participants from each professional group in health centers and hospitals was allocated using sampling proportionate to size technique.

In the case of Addis Ababa, a central referral hospital and another regional hospital was randomly selected and the remaining size of 122 study participants after the selection of 20 health extension workers was proportionally allocated to size of health care providers in these hospitals. In each selected hospital in Addis Ababa, the size was proportionally allocated by type of profession to recruit study participants from each health professional group.

Data collection questionnaire was developed after reviewing pertinent researches done on the research issue (attached as supplementary material). The questionnaire has five sections including, identification particulars of health care providers (HCPs), the socio-demographic characteristics of study HCPs, knowledge, attitude, and practice of them in measuring vitamin D status, diagnosis of deficiency and management and treatment of its conditions. The tool was pilot tested in a similar context where the actual study was not conducted. A total of six graduate students in public health nutrition with a research experience were recruited as data collectors and three MPH graduates were their supervisors. A 3 days training was provided for the field staffs which was followed by intensive supervision during data collection. An Open Data Kit (ODK) template was used for data collection which allows offline data collection. Data were submitted to the cloud server whenever there is access to the internet. Data were exported to Stata version 14 software for cleaning and analysis.

### Data analysis

The study population was described using socio-demographic characteristics. Besides, composite indicators were calculated to measure the level of knowledge, attitude, and practice. The different dimensions of knowledge, attitude, and practice were described using tables and figures. In addition to this, the association of various attributes of health care workers with their practice in measuring vitamin D status, diagnosis of deficiency, and management and treatment of health consequences was assessed using the Chi-square test. Besides, odds ratio along with the 95% confidence interval in binary logistic regression was used to measure the strength, direction, and significance of association between socio-demographic characteristics and the practice of health care workers.

## Results

From a total of 424 recruits, 405 participated in this study resulting in a 95.5% response rate. The general characteristics of the study participants are presented in Table 1. A nearly equal proportion of study participants were recruited from the three ecologies. A little over half 228 (56.3%) of the health workers were females while the overwhelming majority 375(92.3%) were involved in clinical practice. Nurses constituted 152 (37.5%) while health extension workers were 97(24.0%) and physicians 85(21.0%).

**Table 1:** Characteristics of health care providers involved in the knowledge, attitude, and practice study on adult vitamin D deficiency in three ecologic zones of Ethiopia, July 2019

Variable	Response categories	Frequency	Percent
Study area:	Addis Ababa	140	34.6
	Highland	135	33.3
	Lowland	130	32.1
Age group:	19–25	71	17.3
	25–29	192	46.7
	30–39	112	27.3
	40 and over	36	8.8
Sex of the respondent:	Male	177	43.7
	Female	228	56.3
Role in the facility:	Leader/Program officer	30	7.4
	Clinician	375	92.3
Profession:	Physician	85	21.0
	Nurse	152	37.5
	Health officer	71	17.5
	Health Extension Worker	97	24.0
Service years:	Less than 5	186	45.3
	5–9	134	32.6
	10–14	56	13.6
	15 plus years	35	8.5
Special focus on Vit D in your college training?	Yes	367	90.6
	No	38	9.4
In-service training on Vitamin D after graduation:	Yes	7	1.7
	No	398	98.3

A little less than half 186 (45.3%) of participants worked for less than 5 years in their professional career. More than nine in ten of the participated health care workers 367 (90.6%) indicated that they have a pre-service training on vitamin D although only 7(1.7%) of them reported an in-service training on vitamin D level measurement, diagnosis of deficiency, and management and treatment of its health consequences.

**Fig. 1:**
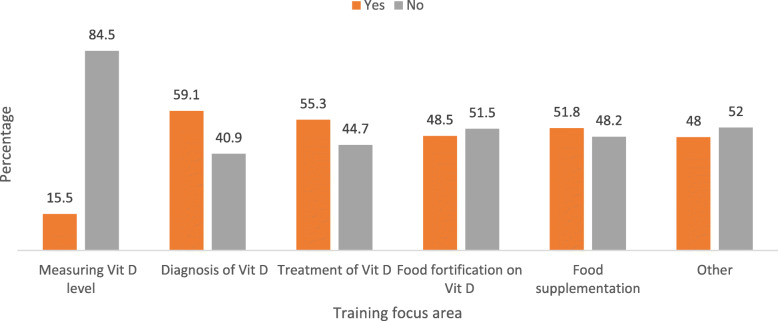
University/College training focus areas on vitamin D deficiency for health care providers, July 2019

As shown in Fig. 1, the main focus areas related to vitamin D during the pre-service training were diagnosis of deficiency for 59.1% of providers, treatment of deficiency-related health conditions according to 55.3% of health care workers, food fortification on vitamin D for 48.5% respondents and food supplementation according to 51.8% of providers.

### Knowledge of health care professional on adult vitamin D deficiency

As shown in Table 2, nearly a quarter of health care providers 95(23.5%) believe that dietary sources are sufficient to maintain vitamin D levels and only 11 (2.7%) think that mid-day is the best time to get vitamin D from sun exposure. However, a little more than half 207(51.1%) of the respondents revealed that 30 min is the optimal duration of sun exposure per day to get vitamin D. The proportion of health care workers who mentioned teenagers, adults and elderly people are risky population groups for vitamin D deficiency was 6 (1.5%), 6 (1.5%) and 28(6.9%), respectively. Regarding the experience of HCWs on measuring vitamin D level, identifying deficiency and management and treatment of ill health consequences only 10(2.5%), 38(9.4%), 39(9.6%) and 25(6.2%) of the health care workers felt that their competency in measuring level, diagnosing deficiency, management, and treatment of ill health consequences and all of those skills mentioned above respectively was good. Overall, only 210 (51.1%) of the study health care workers had a good knowledge score in this study.

**Table 2:** Knowledge of health care providers on adult vitamin D level measurement, diagnosis of deficiency and its management and treatment in three ecologic zones of Ethiopia, July 2019

Variables	Response categories	Frequency	Percentage
Dietary sources are sufficient to maintain Vit D levels:	Yes	95	23.5
	No	310	76.5
Best time of day to get vitamin D from the sun:	Morning	391	96.54
	Mid-day	11	2.7
	Afternoon	3	0.74
Optimal duration of sun exposure/day to get Vit D:	< 30	112	27.7
	30	207	51.1
	> 30	86	21.2
Age groups at more risk of vitamin D deficiency:	U5C	365	90.1
	Teenagers	6	1.5
	Adults	6	1.5
	Elderly People	28	6.9
Personal assertion on competency of measuring Vit D level:	Poor	287	70.9
	Fair	108	26.6
	Good	10	2.5
Personal assertion on competency of Vit D deficiency diagnosis:	Poor	173	42.7
	Fair	194	47.9
	Good	38	9.4
Personal assertion on Vit D management and treatment:	Poor	193	47.7
	Fair	173	42.7
	Good	39	9.6
Personal assertion on measuring Vit D level, vit D deficiency diagnosis and management and treatment:	Poor	195	48.2
	Fair	185	45.7
	Good	25	6.2
Knowledge Score:	Poor	195	48.2
	Good	210	51.8

**Fig. 2:**
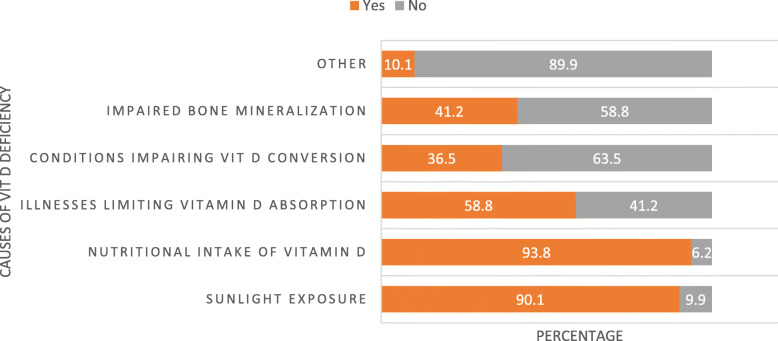
Causes of adult vitamin D deficiency as reported by health care providers in three ecologic zones of Ethiopia, July 2019

Figure 2 showed inadequate sunlight exposure, the inadequacy of nutritional intake on food items rich in vitamin D, illnesses limiting vitamin D absorption, conditions impairing vitamin D conversion and impaired bone mineralization was reported as a cause of vitamin D deficiency by 90.1, 93.8, 58.8, 36.5 and 41.2% of the health workers included in this particular study.

**Fig. 3:**
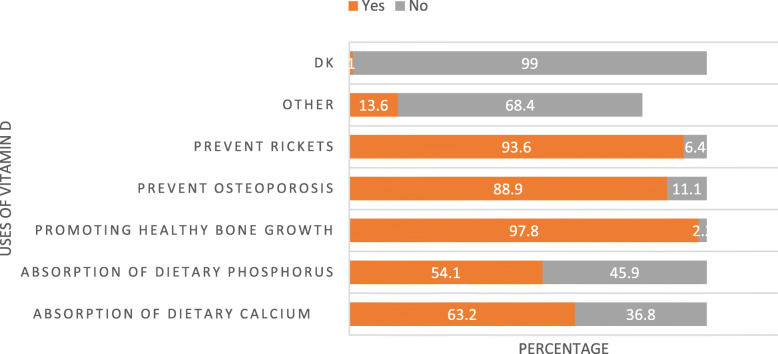
Uses of Vitamin D as reported by health care providers in three ecologic zones of Ethiopia, July 2019

On the other hand, Fig. 3 revealed that promotion of healthy bone growth, prevention of rickets, osteoporosis, and absorption of dietary calcium and phosphorous were reported as the uses of vitamin D by 97.8, 93.6, 88.9, 63.2 and 54.1% of health care workers participated in this study respectively.

**Fig. 4:**
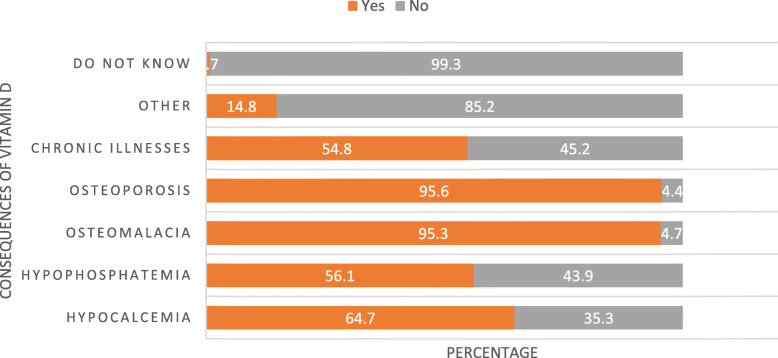
Health consequences of vitamin D deficiency as reported by health care providers in three ecologic zones of Ethiopia, July 2019

The proportion of HCWs who described ill-health consequences of vitamin D such as osteoporosis, osteomalacia, hypocalcemia, hypophosphatemia and chronic illnesses was 95.6, 65.3, 64.7, 56.1, and 54.8%, respectively (Fig. 4).

**Fig. 5:**
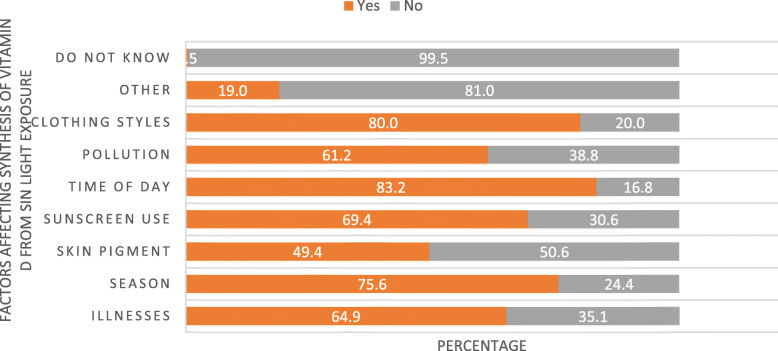
Factors affecting synthesis of vitamin D from sunlight exposure as reported by health care providers in three ecologic zones of Ethiopia, July 2019

When health care workers were asked about factors affecting the synthesis of vitamin D from sunlight exposure, they indicated time of day (83.2%), clothing styles (80.0%), season (75.6%), sunscreen use (69.4%), illnesses (64.9%) and pollution (61.2%) as main ones influencing syntheses (Fig. 5).

**Fig. 6:**
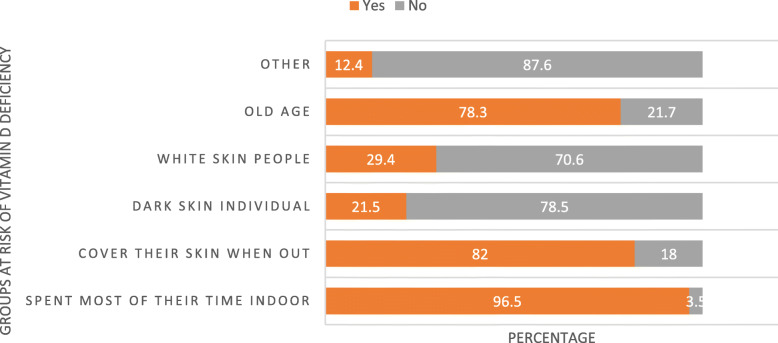
Population groups at more risk of vitamin D deficiency as reported by health care providers in three ecologic zones of Ethiopia, July 2019

According to health workers, people who spent most of their time indoor (96.5%), cover their skin when going out (82.0%), old age persons (78.3%) and white skin people (29.4%) were the main population groups at more risk of vitamin D deficiency (Fig. 6).

### The attitude of health care workers on adult vitamin D deficiency

Table 3 shows that 174(43.0%) of HCWs thought vitamin D deficiency is a public health problem and 183(45.2%) recommended universal screening to identify deficiency. However, only 16(4.0%) believe that there is an adequate laboratory investigation to diagnose vitamin D deficiency in the study area. Besides, only 45(11.1%) of study participants mentioned that vitamin D supplementation is adequate in Ethiopia. Moreover, a few 66(16.3%) thought that they are adequately aware of the prevention of vitamin D deficiency and treatment of associated diseases. On the other hand, the majority 394(97.3%) of them reported that there is a need for community sensitization on the deficiency of vitamin D. However, only 13(3.2%) of them indicated that the ministry of health or regional health bureaus has given adequate attention to the issue under the caption. Though 289(70.6%) of respondents believed that vitamin D deficiency needs an easy and less costly intervention only a few 7(1.7%) of them believed that health care providers are adequately trained on measurement of vitamin D level, diagnosis of its deficiency, and management and treatment of its ill-health consequences. The overall positive attitude score in this study was estimated to be 216 (63.5%).

**Table 3:** Attitude of health care providers towards adult vitamin D level measurement, diagnosis of deficiency and its management and treatment in three ecologic zones of Ethiopia, July 2019

Vitamin D Deficiency is a public health problem:	Yes	174	43.0
	No	231	57.0
There should be universal screening for vitamin D deficiency:	Yes	183	45.2
	No	222	54.8
There is an adequate laboratory investigation to diagnose Vit D def:	Yes	16	4.0
	No	342	8.4
	DK	47	11.6
There is an adequate Vitamin D supplementation in Ethiopia?	Yes	45	11.1
	No	303	74.8
	DK	57	14.1
Healthcare practitioners adequately aware of prevention and Rx:	Yes	66	16.3
	No	339	83.7
There is a need for community sensitization on deficiency of vitamin D:	Yes	394	97.3
	No	11	2.7
MOH/RHB gave adequate attention to Vitamin D deficiency:	Yes	13	3.2
	No	371	91.6
	DK	21	5.2
Vitamin D deficiency needs an easy and less costly intervention:	Yes	289	70.6
	No	119	29.4
Health care providers are adequately trained on vitamin D:	Yes	7	1.7
	No	385	95.1
	DK	13	3.2
Attitude Score:	Poor	150	36.5
	Good	261	63.5

*DK* do not know, *Rx* treatment, *RHB* regional health bureau, *MOH* ministry of health

### Practice of health care providers on adult vitamin D deficiency

Only 46(11.4%) of health care workers reported that they have diagnosed adult patients for vitamin D deficiency in their professional career, of those 26(56.5%) of them diagnosed 1–3 patients of any age on an average working month (see Table 4). When asked on mechanisms of diagnosis 41(89.1%), did it by making clinical examination, 26(56.5%) measured serum vitamin D and 21(45.7%) used a combination of them. Moreover, 26(56.5%) revealed that they measured a concentration of 25 OH to assess vitamin D status.

**Table 4:** Practice of health care providers on adult vitamin D level measurement, diagnosis of deficiency and its management and treatment in three ecologic zones of Ethiopia, July 2019

Variables		Freq	Per
Have you ever diagnosed adult patients for vitamin D deficiency?	Yes	46	11.4
	No	359	88.6
How many patients of any age do you diagnose in an average month?	1–3	26	56.5
	4 plus	20	43.5
Clinically:	Yes	41	89.1
		5	10.9
Measuring serum Vitamin D:	Yes	26	56.5
		20	43.5
Both:	Yes	21	45.7
		25	54.4
Did you measure concentration of 25 OH vitamin D to assess vitamin D status:	Yes	26	56.5
	No	20	43.5
Did you prescribe a vitamin D supplement for adults recently?	Yes	50	12.4
	No	355	87.6
Do you prescribe vitamin D supplements for pregnant women recently?	Yes	3	0.7
	No	402	99.3
Is there any guideline to recommend for vitamin D supplementation?	Yes	13	3.2
	No	392	96.8
Have you ever given calcium supplement for adults to prevent the ill consequences?	Yes	77	19.0
	No	328	81.0
Do you usually counsel your adult patients about the importance of Vit D?	Yes	64	15.8
	No	341	84.2
Do you advise adult patients about the source of vitamin D?	Yes	89	22.0
	No	316	78.0
Do you ask your adult patients about adequate sunlight exposure?	Yes	53	13.1
	No	352	86.9
Do you counsel your adult patients on the need for sunlight exposure for Vit D?	Yes	86	21.2
	No	319	78.8
Do you ever attend any on the job training/workshop on Vit D deficiency?	Yes	26	6.4
	No	379	93.6
Practice Score:	No	216	52.6
	Yes	195	47.4

Besides, only 50(12.4%) of study HCWs prescribed vitamin D supplementation for adults recently. Vitamin D supplementation for pregnant women was prescribed by only 3(0.3%) of the health care workers. Also, 13(3.2%) of health care workers used guidelines to recommend vitamin D supplementation. This study also revealed that 77(19.0%) of health care workers have given calcium supplement for adults to prevent the ill consequences of vitamin D deficiency in their full-service years. In addition to this, 64(15.8%) of health care providers usually counseled their adult patients about the importance of vitamin D deficiency. Meanwhile, 89(22.0%) of health care workers have advised adult patients about the source of vitamin D and 53(13.1%) of them asked their patients about the adequacy of sunlight exposure for vitamin D. Moreover, 86(21.2%) of health care workers counseled their adult patients on the need for sunlight exposure to get vitamin D. However, only 26 (6.4%) of health care providers received an on-job training or workshop on diagnosis of deficiency and treatment of vitamin D.

### Factors associated with the practice in measuring vitamin D status, diagnosis of deficiency, and management and treatment of its ill health consequences:

The practice in measuring vitamin D status, diagnosis of deficiency, and management and treatment of ill-health conditions is significantly different among providers deployed in the three ecologies, which is higher among health workers working in Addis Ababa followed by those working in the rural highland district (Table 5). Male providers had a significantly better practice compared with females and as the age of the provider increases the practice level is improving. Obviously, clinicians have a better practice compared with those assigned in leadership and health promotion positions though the difference is not statistically significant. The practice level was significantly better among physicians and health officers, whose trainings have more emphasis on clinical orientation. Moreover, those workers who worked for several years had a significantly better practice level compared with the junior ones. In addition to this, health workers who have good knowledge and attitude had better practice in measuring vitamin D level, diagnosis of deficiency, and management of ill-health consequences.

**Table 5:** The cross-tabulation of different characteristics of health care providers with the practice of them on measuring level, diagnosing deficiency and its management and treatment in three ecologic zones of Ethiopia, July 2019

Variables	Practice: #(%)		*p*-value
	No	Yes	
Study area:			
Addis Ababa	40(28.6)	100(71.4)	0.000
Highland	82(60.7)	53(39.3)	
Lowland	94(72.3)	36(27.7)	
Sex:			
Male	78(44.1)	99(55.9)	0.001
Female	138(60.5)	90(39.5)	
Age group:			
Less than 25	48(67.6)	23(32.4)	0.000
25–29	112(58.3)	80(41.7)	
30–39	44(39.3)	68(60.7)	
40 and over	12(33.3)	24(66.7)	
Role:			
Clinician	197(52.5)	178(47.5)	0.254
Program officer	19(63.3)	11(36.7)	
Profession:			
Physician	17(20.0)	68(80.0)	0.000
Nurse	99(65.1)	34(34.9)	
Health officer	38(53.5)	33(46.5)	
Health Extension Worker	62(63.9)	35(36.1)	
Service year:			
Less than 5 years	104(55.9)	82(44.1)	0.063
5–9 years	70(52.2)	64(47.8)	
10–14 years	31(55.4)	25(44.6)	
15 plus years	11(31.4)	24(68.6)	
Knowledge			
Poor	125(64.1)	70(35.9)	0.000
Good	91(43.3)	119(56.7)	
Attitude:			
Poor	93(62.0)	57(38.0)	0.004
Good	123(47.1)	138(52.9)	

Table 6 showed the binary logistic analysis to identify health care workers’ characteristics associated with their practice in measuring vitamin D level, diagnosing deficiency, and management and treatment of ill-health consequences. The odds of vitamin D service provision practice was AOR = 6.87: 95% CI (3.57, 13.21) times statistically significantly higher among health care workers deployed in Addis Ababa and AOR = 2.20: 95% CI (1.23, 3.92) times statistically significantly higher in the rural highland compared with those health care workers deployed in the rural lowland area. The likelihood of male health care workers’ practice in the provision of vitamin D related service was AOR = 1.26: 95% CI (0.71, 2.22) times statistically significantly higher compared with females. However, the difference in the odds of practice in vitamin D service provision vanished when it is adjusted for other socio-demographic factors.

**Table 6:** The binary logistic regression of different characteristics of health care providers with the practice of them on measuring level, diagnosing deficiency and its management and treatment in three ecologic zones of Ethiopia, July 2019

Variables	Crude OR (95% CI)	*p*-value	Adjusted OR (95% CI) ^a^	*p*-value
Study area:				
Addis Ababa	6.53(3.84, 11.10)	0.00	6.87(3.57, 13.21)	0.00
Highland	1.69(1.01, 2.83)	0.04	2.20(1.23, 3.92)	0.00
Lowland	1.00		1.00	
Sex:				
Male	1.95(1.31, 2.90)	0.01	1.26(0.71, 2.22)	0.43
Female	1.00		1.00	
Age group:				
19–25	1.00		1.00	
25–29	1.49(0.84, 2.65)	0.17	1.03(0.51, 2.08)	0.93
30–39	3.23(1.73, 6.03)	0.00	1.98(0.82, 4.80)	0.13
40 and over	4.17(1.78, 9.79)	0.00	1.23(0.20, 7.52)	0.82
Role:				
Clinician	1.56(0.72, 3.37)	0.26	4.26(1.48, 12.25)	0.00
Programmer	1.00		1.00	
Profession:				
Physician	7.09(3.61, 13.90)	0.00	1.53(0.53, 4.45)	0.43
Nurse	0.95(0.56, 1.61)	0.85	0.42(0.20, 0.91)	0.03
Health officer	1.54(0.82, 2.87)	0.18	0.50(0.20, 1.25)	0.14
HEW	1.00		1.00	
Service year:				
< 5 years	1.00		1.00	
5–9 years	1.16(0.74, 1.81)	0.52	1.06(0.59, 1.92)	0.85
10–14 years	1.02(0.56, 1.87)	0.94	0.91(0.39, 2.13)	0.83
15 plus years	2.77(1.28, 5.98)	0.01	1.80(0.33, 9.72)	0.49
Knowledge:				
Poor	1.00		1.00	
Good	2.34(1.57, 3.48)	0.00	1.96(1.19, 3.23)	0.00
Attitude:				
Negative	1.00		1.00	
Positive	1.83(1.22, 2.76)	0.00	2.30(1.40, 3.78)	0.00

*HEW* Health Extension Worker

^a^Adjusted for study area, sex, age group, role, profession, service year, knowledge and attitude

Besides, the likelihood of clinicians’ practice in the provision of vitamin D service was AOR = 4.26: 95% CI (1.48, 12.25) times statistically significantly higher compared with those working in leadership and health program positions. The difference in the odds of competency in vitamin D service provision among different professional groups vanished when it is controlled for other socio-demographic factors. The same is true for the service year of health care workers.

On the other hand, the adjusted odds of practice in vitamin D service provision was AOR = 1.96: 95% CI (1.19, 3.23) times statistically significantly higher among those health care workers who have a good knowledge on vitamin D related service compared with those with poor knowledge. Besides, the likelihood of better practice in vitamin D service provision was AOR = 2.30: 95% CI (1.40, 3.78) times statistically significantly higher among those health care workers with a positive attitude in vitamin D service provision compared with those who did have a negative attitude.

## Discussions

The current study revealed that only a little over half of HCWs in Ethiopia have good knowledge in measuring adult vitamin D levels, diagnosis of deficiency, and management of ill-health consequences. About one in four of study participants thought dietary food sources are good enough for vitamin D, only about 3% knew mid-day as the best time to sun exposure for vitamin D and 51.1% knew the ideal duration of sun exposure of 30 min per day. Teenagers, adults and elderlies, as more risky population groups for vitamin D deficiency, were mentioned by 1.5, 1.5 and 6.9% of HCWs respectively. On the other hand, a little lower than two-third of participants has a positive attitude towards adult vitamin D service and 43.0% think adult vitamin D deficiency is a public health problem. Besides, HCWs with overall good practice of adult vitamin D service provision were 47.4, and 11.4% of them tried diagnosis of its deficiency among adult patients, and 12.4% prescribed supplementation for adults. Moreover, adult vitamin D service good practice was higher among health workers in Addis Ababa and highland areas compared with those working in lowlands. Male HCWs were more likely to have a good practice on adult vitamin D service compared with females. Clinicians were more likely to have a good practice in adult vitamin D service compared with those working in leadership and health program positions. Good practice in adult vitamin D service provision was higher among HCWs who have a good knowledge and positive attitude compared with their counterparts.

The study revealed gaps in the knowledge of HCWs on adult vitamin D deficiency. This finding is similar to the findings of similar studies done in Australia and New Zealand [9, 10] while it is lower than the finding in Saudi Arabia [23]. This finding is really critical in the sense that if HCWs themselves have a knowledge gap then it will be difficult to manage and treat health conditions due to vitamin D deficiency which will exacerbate ill-health conditions among adults [8, 9, 24].

Research shows that sunlight is an important and best source of vitamin D because it produces vitamin D in the skin that may last twice as long in the blood compared with ingested vitamin D [25]. According to Vitamin D Council, dark-skinned individuals need hours of sunlight exposure than light-skinned people. Only half time is needed to produce sufficient amount of Vitamin D before burning the skin. At least 25% of skin surface has to be exposed. UVB can’t penetrate glass windows and there is no overdose of Vitamin D from overexposure to sunlight [26]. However, in the current study, only half of the HCWs identified sunlight exposure as the most common source of vitamin D. The rest of them indicated sources other than sunlight. Because UV radiation is the main risk factor for skin cancer, there is a debate on advice against or recommend sun exposure and physicians are usually confused on encouragement or discouragement of sun exposure as a result some physicians do not recommend their patients to sunlight. But generally, there is a growing evidence and knowledge that suggested insufficient exposure to UV radiation is associated with risks to overall health and even shortens life expectancy [27].

The study also indicated population groups with limited exposure to sunlight as being at high risk of vitamin D deficiency, including individuals who spend most of their time in indoor activity and old age persons. Studies indicated that dark skin color is considered to be a major factor in vitamin D deficiency [28] and vitamin D concentration differ by color with dark skin individuals producing less vitamin D compared with light skin individuals in the same amount of sunlight exposure [29, 30]. But in the current study dark skin was identified as a risk factor for vitamin D deficiency by only one-quarter of the respondents which is similar to a study done in Australia [19].

Another most common confusion among participants was the appropriate time of the day for sun exposure. More than half of the respondents reported morning as an appropriate time for sunlight exposure. Only a very minimal portion (2.7%) of HCWs were able to identify mid-day as an appropriate time for sun exposure to get vitamin D. However previous studies in the area ruled out that mid-day between 10:00 am and 3:00 pm is an appropriate time for sunlight exposure [25]. This implied that HCWs in Ethiopia recommended a wrong time for sunlight exposure to get vitamin D from UV.

The study also revealed that a little less than two-third of study participants have a positive attitude towards adult vitamin D service provision in Ethiopia which has a lot of implication for capacity building of HCWs as it may lead to misdiagnosis of deficiency by frontline health workers which leads to complications of health conditions due to shortage of the mineral considered in this study [8, 9].

The study also showed a lot of gap in practice of health care workers in the provision of adult vitamin D service. It is found that a very small percent of HEWs have ever diagnosed vitamin D deficiency throughout their professional careers. A study done in America also showed physicians do not often consider vitamin D deficiency in their adult patient management and it is very common for them to misdiagnose it [31]. The reason for this may be health care professional often think that their patients in Ethiopia might have adequate sunlight exposure as they are living near the equator. But most of the time, especially aged people, become home bounded and do not get adequate sunlight which predispose them for vitamin D deficiency [32]. This might be a possible reason for Ethiopian health care workers to ignore vitamin D deficiency in their management of chronic diseases for adults. Although there is a controversy on the cutoff point on level of 25-hydroxyvitamin D, to define vitamin D deficiency, the US Endocrine Society defines vitamin D as 25-hydroxyvitamin D level below 20 ng/ml (50 nmol/L) [33]. In line with this, the study done in Ethiopia in 2013 revealed the prevalence of vitamin D deficiency among adult women was 84.2% [4]. The small percentage of HCWs who were able to diagnose vitamin D deficiency throughout their career life indicated a missed opportunity in the diagnosis of vitamin D deficiency for patients coming to health facilities. This could be attributed to the poor in-service training that hasn’t been given much attention for adult vitamin D deficiency in Ethiopia as documented in this study.

This study also showed a small proportion of HCWs ask and counsel their adult patients about sunlight exposure as documented by different studies that also showed physician to miss asking this question [18, 19]. This may be due to either lack of knowledge or minimal attention of health care professionals towards vitamin D or in some cases sunlight exposure is believed to be related with skin cancer. Physicians encourage their patients to use sunscreen when they go out and in some cases they counsel to avoid sunlight exposure because of fear of the risk of skin cancer [18, 19].

There is also a major difference and confusion on optimal time and duration of sunshine exposure as evidenced in this study. The study conducted in Saudi Arabia also shows that physicians have different opinions, knowledge, and practice on adult vitamin D [23]. This difference might be attributed to the use of different guidelines and source of information which implies the Ethiopian government to prepare its own national guideline for adult vitamin D deficiency management.

The study showed that health care workers working in Addis Ababa and rural highland areas in Ethiopia have a better practice of adult vitamin D service compared with those living in rural lowland areas which could be related to the physicians misunderstanding that people living in arid lowland areas have a better access to sunlight exposure though studies in the middle East indicated the opposite [12, 34].

Generally, since this is the first study done in country, it is believed that, it will give a baseline information on knowledge, attitude, and practice of health care workers on adult vitamin D deficiency which will be used as a reference for future studies.

### Strengths and limitations of the study

Its large sample size, inclusion of respondents from different categories of health workers (health extension workers, nurses, health officers, general practitioners, and specialists), its coverage of different ecological zones (rural and urban areas, highland, midland, and lowland areas) and low none-response rate could be considered as strengths of the study.

The main limitation of the study emanates from its study design in which we cannot establish a causal relationship. On the other hand, though studies suggest cutoff point to dichotomize knowledge, attitude, and practice, there is no standard cut-off point to classify as “good” and “bad” Or “negative” and “positive”. The other limitation might be attached to the social desirability bias whereby respondents might report what is presumably acceptable by the research team that may influence the magnitude of knowledge, attitude, and practice.

## Conclusions

The study identifies low level of knowledge, attitude, and practice of health care workers on adult vitamin D status measurement, diagnosis of deficiency, and management and treatment of its health consequences. Moreover, the study reveals that health care workers with good knowledge and a positive attitude have a better practice of adult vitamin D service provision. Besides, female health workers, those working in rural lowland areas and HCWs with non-clinical position were associated with the poor practice of adult vitamin D service provision.

We recommend to have rigorous and continuous in-service trainings on adult vitamin D deficiency for health care workers in different levels of health facilities assuming different positions. Also, special attention should be given to female health care workers, health workers assigned in rural areas where accesses to updated information and technologies are limited, health professionals working in health facilities other than hospitals, and low and mid-level health professionals. To reach at a consensus and avoid confusion regarding the definition, investigation of vitamin D deficiency and its treatment procedure, the ministry of health and partners working on micro-nutrient supplementation should give better attention to design appropriate national guidelines for adult vitamin D deficiency management.

## Data Availability

The datasets used during the current study are available from the corresponding author on reasonable request.

## Abbreviations

HCWs: Health care workers
OR: Odds ratio
AOR: Adjusted Odds Ratio
UVB: Ultraviolet B
HCPs: Health care providers
ODK: Open data kit
DK: Don’t know
Vit D: Vitamin D
MOH: Ministry of Health
VD: Vitamin D
